# How to Increase Employees’ Proactive Vitality Management? Testing the Effect of a Training Intervention

**DOI:** 10.3390/ijerph192315898

**Published:** 2022-11-29

**Authors:** Alexandra Bălăceanu, Delia Vîrgă

**Affiliations:** Department of Psychology, West University of Timisoara, 300223 Timișoara, Romania

**Keywords:** proactive vitality management, intervention, work-related strategies, micro-breaks, online

## Abstract

Drawing on the Job Demands-Resources theory, we investigate the effectiveness of an online intervention based on training in energy management strategies using an experimental design. The intervention focused on creating awareness about the importance of energy in completing tasks, shaping the present state, and proactively identifying valuable strategies to manage vitality during work. Additionally, we expected an increase in work-related strategies (i.e., setting a new goal) and a decrease in micro-breaks (i.e., mental and physical). Participants were enrolled voluntarily in the intervention and randomly assigned to the experimental group (N = 42) and the control group (N = 44). Results of the ANCOVA showed that, in the experimental group, the intervention positively impacted changes in proactive vitality management. Furthermore, the results indicated that the participants from the experimental group used fewer physical micro-breaks after the intervention. Additionally, after the training and weekly level, the results showed a decrease in work-related strategies and physical micro-breaks in the experimental group. Thus, organizations could facilitate employees to learn to engage in different energy management strategies according to their preferences.

## 1. Introduction

Organizations nowadays need proactive employees to rapidly adapt to demanding contexts. More than ever, employees must be responsible for their work and manifest proactive behaviors to adapt, improve, and innovate [1]. In this context, human energy is an essential resource for organizations to operate successfully and adapt to the new challenges related to the work environment. Based on the Job Demands-Resources (JD-R) theory [2], Op den Kamp and collaborators [3] proposed proactive vitality management (PVM) as a new form of individual strategy. PVM represents an individual’s “goal-oriented behavior aimed at managing physical and mental energy to promote optimal functioning at work” [3] (p. 10). For example, an employee can choose to manage their energy by going for a walk, meditating, or drinking a cup of coffee. Employees can replenish energy, an essential but limited resource, by listening to their needs [3,4]. PVM could also increase resources that allow employees to prevent occupational stress [5] and adapt to new demands [6]. In this vein, increasing employees’ PVM could be a strategic investment for organizations.

Previous studies have investigated micro-breaks and work-related strategies as ways that employees manage (replenish and increase) their energy during work hours [7,8]. Work-related strategies are supposed to be techniques that “employees use while doing their work to manage their energy” (e.g., switching tasks, making a to-do list; p. 31) [7]. Micro-breaks are part of energy management strategies that are not directly related to work. However, employees could replenish energy levels in the work program by having a snack, doing some form of physical activity, or listening to music. We propose that different micro-breaks (e.g., having a snack) and work-related strategies (e.g., making a to-do list or learning something new) could have a different impact on increasing the level of PVM at work. Therefore, the present study aims to test if employees’ PVM rises due to the intervention, which combines work-related strategies and micro-breaks during the work program.

Our study contributes to the literature in several ways. From a theoretical perspective, we bring evidence for the essential role of PVM in the JD-R theory as a self-initiated behavior at work directed at sustaining rather than replenishing employees’ energy levels. We demonstrate the effectiveness of a pioneering intervention in which employees use different energy management strategies to maintain optimal energy levels during working hours. From a practical perspective, our study offers organizations an overview of actions that can be delivered to employees to enhance their PVM. We provide employers with the tools to train their employees on better energy management to function optimally in the workplace. Additionally, the results will help organizations recognize the benefits of re-energizing activities at work.

### 1.1. Theoretical Background

Because PVM is a new concept in the positive organizational behavior literature, only a few studies have investigated the relations between PVM and different outcomes [9,10,11]. Although empirical evidence for the benefits of PVM is accumulating, no studies have investigated the effectiveness of an intervention to improve this proactive behavior. Our paper addresses this gap by elaborating on and testing the first intervention developed to increase PVM. This intervention builds on previous theoretical premises and positive results of interventions designed to improve proactive behavior, i.e., increased personal initiatives; [12,13]. These interventions have mostly drawn on German action theory [14], which states that goal-oriented behaviors, such as proactive behaviors, involve the action sequences of developing goals, collecting information, generating and executing plans, and processing feedback. Therefore, an intervention that increases employees’ commitment to the goals and delivers quality information is expected to make employees more proactive [15]. As already stated, all previous interventions have focused on other proactive behaviors. PVM is a less investigated proactive behavior due to its novelty in the literature focused on JD-R theory.

The JD-R theory [2] indicates that all job characteristics can be divided into job resources and demands. Job resources are work-related aspects that reduce job demands, help achieve job goals, and stimulate learning, personal growth, and development [2], such as job autonomy and supervisor support. Job demands are characteristics of a job that need effort (physical and/or psychological), implying certain physiological and/or psychological costs [2], such as work overload and high work pressure. The JD-R theory introduces a dual pathway related to well-being—a health impairment and motivational process that are impacted not only by each type of job characteristic but also by the interaction between job demands and resources. Besides including personal resources, the JD-R theory was complemented recently by integrating individual strategies, which are “methods or plans that people choose to achieve a goal or solve a problem, which generally involves some planning or marshaling of resources for their most efficient and effective use” [16] (p. 1106). Demerouti et al. [17] proposed that individual strategies could help undermine the effects of job strain or potentiate the favorable effects of resources. In what follows, we consider PVM in the category of individual strategies that maximize the beneficial impact of job characteristics (i.e., self-regulation) on the motivational process, specifically on employee engagement and performance.

Based on JD-R theory, all types of individual strategies (i.e., strengths use, job crafting, PVM) reflect self-initiated actions employees take to effectively use existing resources, reflecting on their well-being and performance at work. However, the resources these strategies capitalize on can have differing sources. For example, focusing on utilizing internal resources (i.e., strengths) to achieve better functioning at work. On the other hand, job crafting and PVM encompass the marshaling of external resources, either organization- (i.e., job crafting) or non-organization-dependent (i.e., PVM). Thus, PVM is a self-initiated behavior that mobilizes employees’ resources to conserve their energy and diminish job demands, leading to higher work engagement levels when resources are available [3].

Furthermore, engaged employees will be more likely to manage their energy to perform well, which is the ultimate goal of PVM. Motivated and engaged employees have energy that allows them to gain more resources and achieve positive outcomes through PVM. In contrast, mentally and physically exhausted employees are deprived of energy; consequently, they cannot proactively manage a resource unavailable to them. Moreover, the JD-R theory indicates that employees exposed to high job demands are less likely to engage in recovery activities after work and less able to recuperate [18]. The same logic can apply during working hours from an energy management perspective. Thus, when employees are stressed, they cannot manage their energy at work. In this context, they become passive and reactive rather than proactive [19]. Therefore, as self-regulation behaviors, energy management strategies must be manifested before the exhaustion phase is installed to ensure that resources are available to be carefully managed. By proactively engaging in energy management strategies (i.e., work-related strategies, physical and mental micro-breaks), employees could have the energy to create more resources. Thus, the impact of job demands can be diminished, and employees’ health can be protected. Additionally, PVM is linked with employees’ mental health through work engagement [11] and performance via work engagement [10]. Being aware of one’s energy is an advantage because, in this way, individuals can use different energy management strategies to conserve or regain the energy to perform their tasks at work. The next step should be pinpointing the strategies that work for them. Therefore, developing training content on energy management strategies is essential to enhance PVM. In this regard, we formulated the first hypothesis:

**H1.**  *The intervention based on energy management strategies in the workplace positively impacts PVM.*

The literature identified two crucial energy management strategies: micro-breaks and work-related strategies [7,8]. Micro-breaks are linked to lower energy levels than other strategies, which increase energy at work [20]. Thus, micro-breaks are moments of rest from activities at work (such as eating a snack, reading something for fun, or listening to music). These micro-breaks could increase vitality but, in fact, could deplete energy (i.e., drinking coffee and smoking cigarettes, which generate health detriments and outweigh the health benefits of taking frequent breaks). De Bloom et al. [21] differentiated micro-breaks into mental and physical breaks. Mental micro-breaks refer to short breaks during which individuals engage in activities and focus on other things during working hours (e.g., listening to music or surfing the internet). Physical micro-breaks are short breaks for physical activities that cover physiological needs (e.g., drinking a glass of water or taking a walk) during the workday. In contrast, work-related strategies include activities that focus on employees seeking meaning (such as learning something new, creating meaning, responding to e-mails, and building positive relationships at work), which is associated with experiencing higher energy and less fatigue [20].

Even if the activities employees engage in are work-related (i.e., energy management strategies), these represent a disruption from the ongoing task, which means a break for the employees. However, this gives individuals a fresh perspective on the previous task, and employees may become more energetic after this break. The difference between the two main energy management categories may lie in the cognitive effort involved. While micro-breaks represent a mental break, work-related strategies require cognitive effort.

Results of previous studies indicate that micro-breaks seem to be linked to recovery moments rather than proactive energy management [20]. This suggests that micro-breaks may be more beneficial as sources of recovery during leisure time (e.g., chatting with a friend on social occasions or going for a walk in the afternoon). In contrast, work-related strategies help employees maintain optimal energy levels during work [3,7,8,22]. In other words, the observed tendency is that work-related strategies are more efficient for managing energy proactively during work compared to the micro-breaks used when energy depletion is installed, and employees need to rest. Therefore, through our study, we want to examine if involving the employees in a training intervention related to different energy management strategies will generate differences in their usability. Thus, we formulated the second hypothesis:

**H2.**  *The use of work-related strategies will increase, and mental and physical micro-breaks will decrease post-test in the experimental group.*

### 1.2. Intervention Design

The intervention design is based on an experimental design with two groups (experimental with intervention vs. control without intervention), with a pre-test–post-test evaluation. Drawing on German action theory [14] and relevant research [23,24], we propose an intervention to increase PVM, which is to be formed as a result of a two-step process due to the proactive component: proactive goal generation and proactive goal striving. Therefore, the German action theory is the central framework of our intervention design, along with the vision-focused intervention approach (based on Strauss et al. [13]). The two-step process includes: (1) Proactive goal generation with envisioning and planning—the employee identifies the task that requires energy to be finalized (envisioning), then strategies will be generated to conserve energy or replenish energy reservoirs (planning). (2) Proactive goal striving with enacting and reflecting—the individual engages in the strategies from the planning step; here, self-regulation is a core aspect (enacting), then the employee would analyze if the strategies used were efficient (e.g., the task is completed, they still feel energized) (reflecting). During the reflecting phase, the individuals can readapt the strategies if they feel their energy is not optimal due to the wrong assessment of the task or the vitality levels before starting the actual task.

As presented above, it is expected that PVM can be trained due to its proactive behavioral nature. Thus, employees will apply the best strategies that generally involve planning or managing resources to achieve work-related goals. Finally, we will also focus on employees’ perceptions of the online training intervention, since it is the first intervention designed to improve PVM.

## 2. Materials and Methods

### 2.1. Participants

The participants were recruited online by posting invitations on different channels (e.g., LinkedIn, Facebook, phone, and in-person meetings) to participate in an online intervention. Only the ones interested contacted us. An a priori power analysis was conducted using G*Power [25] to test the appropriate number of participants for our intervention. The result showed that a total sample of 84 participants was necessary based on the following input parameters: statistical power = 0.85, medium effect size d = 0.5, α = 0.05, and the number of groups = 2 (experimental vs. control).

**Figure 1:**
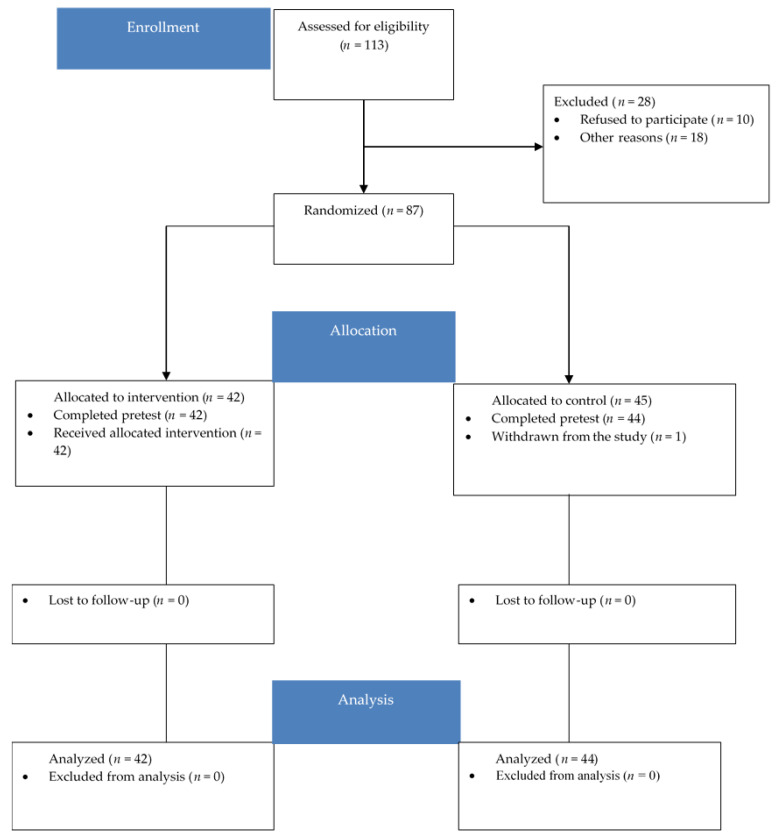
CONSORT Flow Diagram [26].

Thus, we recruited an initial pool of 113 participants, from which 87 full-time employees participated in the intervention, representing a response rate of 77%. They worked in diverse industries: automotive, information technology (IT), consultancy, human resources, and health. Out of the 87 participants, 1 voluntarily withdrew from the control group during the intervention. Therefore, the final sample consisted of 86 participants. A CONSORT Flow Diagram presents an overview (see Figure 1). The volunteering participants were randomly assigned to one of two groups (experimental and control). The selection criteria included being employed at the moment of selection and being available to dedicate time to training sessions. Additionally, participants needed to be present at work or have ongoing work activity during the study, in order to complete the questionnaires for the monitoring phases. Those on holidays or vacations were not eligible.

The experimental group consisted of 42 participants, and the control group consisted of 44 participants. Women comprised 70% of the training group and 68.2% of the control group. The average age of participants was 32.33 years (SD = 6.84) in the training group, ranging from 24 to 53 years. The control group’s average age was 33.52 years (SD = 8.69), ranging from 21 to 55 years. Regarding education, 27.8% held an undergraduate degree in the experimental group, 4.7% held a vocational degree, and 67.5% held a postgraduate one. On average, participants from the experimental group had 10.26 years (SD = 7.16) of work tenure. Additionally, in this group, participants reported activities in the following domains: 33% in automotive, 19% in IT, 14% in human resources, 14% in health, 12% in consultancy, and 7% in other domains.

In the control group, 75% held an undergraduate degree, 16% held a vocational one, and 9% had a postgraduate one. This group’s work tenure averaged 9.7 years (SD = 8.42). The participants reported activities in the following domains: 27% in human resources, 23% in consultancy, 20% in health, 18% in IT, and 11% in other fields. The differences between the experimental and control groups for demographic characteristics were non-significant (for age, t = 1.62, ns.; for tenure, t = 1.34, ns.).

### 2.2. Measures

At T_1_, in the experimental group, we assessed PVM, work-related strategies, and micro-breaks (i.e., mental and physical). In the control group, we measured PVM. At T_2_, we measured the same variables in both groups, following the same approach as in T_1_. The experimental design implied pre-tests and post-tests for experimental and control groups. In addition, we included a weekly self-monitoring phase for the experimental group, which lasted five weeks. For this, we used the same items from T_1_ and T_2_ but adapted them to the weekly level.

PVM was assessed at T_1_ and T_2_ using the eight-item scale developed by Op den Kamp et al. [3] and validated on the Romanian sample by Bălăceanu et al. [5]. An example item is: “I make sure that I feel energetic during my work” (1 = “totally disagree”; 7 = “totally agree”). The total score indicated a measure of participants’ use of vitality management strategies to promote their work. The internal consistency of the scale was excellent in each measurement moment (experimental group: T_1_, α = 0.88; T_2_, α = 0.90; control group: T_1_, α = 0.80; T_2_, α = 0.95). Since PVM was the primary outcome variable, the minimum detectable effect of the intervention was represented by a significant difference between the value of PVM in the experimental group vs. the control group at T_2_ (post-test evaluation).

To measure work-related strategies within the experimental group at T_1_ and T_2_, we used the scale adapted by de Bloom et al. [21]. The questionnaire includes the most frequently reported strategies and micro-breaks identified by two other studies (i.e., [7,8]. Therefore, five items measure work-related strategies as breaks that relate to the job (e.g., helping a colleague), five items reflect mental micro-breaks (e.g., listening to music), and three items measure physical micro-breaks (e.g., physical activities). The participants used a scale from 1 to 5 to report how frequently they engaged in any of the behaviors (1 = “never” to 5 = “very often”). Because we note that the measures are assumed to be formative rather than reflective, as the combination of its measures defines each construct, a change in any specific measure changes the overall construct, not vice versa. Additionally, we propose that different micro-breaks and work-related strategies do not necessarily have to be correlated. Based on these criteria, in the case of constructs with formative measures, such as energy management strategies, it is inappropriate to compute internal consistency to assess their reliability [27]. Test–retest reliability could be a more appropriate way to assess the reliability of formative constructs if the items are stable over time. Specifically, the correlation between general work-related strategies at T_1_ and T_2_ was *r* = 0.42 (*p* < 0.01). The correlation between mental micro-breaks at T_1_ and T_2_ across the workday was *r* = 0.69 (*p* < 0.01), and the correlation between physical micro-breaks at T_1_ and T_2_ across the workday was *r* = 0.52 (*p* < 0.01). These findings provide initial evidence for the reliability of our measures.

We used the same instruments from T_1_ and T_2_ for the weekly measures, but we adapted them to reflect weekly experiences. An example of an item for work-related strategy is: “This week, I checked my e-mail during my work.”; an example item of a mental micro-break is: “This week, I listened to music during my work.”; an example item of a physical micro-break is: “This week, I did some physical activity, including walking or stretching during my work time.”. Participants were asked how often they had used each of the 13 strategies during the week (for 1 = “never” to 5 = “very often”).

As a manipulation check, we measured participants’ perceptions of the training. Thus, we applied a 3-item scale developed for this study to determine if participants’ expectations were met, if the information was interesting, and if they would apply what they learned. Responses were coded on a Likert-type scale, from 1 = “strongly disagree” to 5 = “strongly agree” (α = 0.78). The answers were collected immediately after the training.

### 2.3. Procedure

Before starting the intervention, we completed the formal procedures and obtained the documentation attesting compliance with the ethical aspects of scientific research (no. 24845/31.05.2021). We compared PVM during working hours across two groups of employees from Romania. Participating in this intervention rendered the employees eligible to win a voucher for three individual coaching sessions. We addressed the invitation to participants as an “opportunity for personal growth”. It is expected that proactive individuals might be more likely to participate in this experiment and respond positively to training. Therefore, we used a computer-generated list of random numbers to rule out such selection effects in the process of allocating volunteers to the control and intervention groups, respectively [28]. After the group distribution, the questionnaires from T_1_ were sent to the participants, who had two weeks to complete them. The experimental group received the items that measure PVM, work-related strategies, and micro-breaks, and we also collected data on demographic characteristics.

Additional variables were measured five weeks after the training, representing a self-monitoring phase. The control group received only the PVM measure during the pre-test and post-test. We considered it relevant to collect more data from the experimental group because of their involvement in the training and to avoid learning curves in the control group by exposing them to information about the usage of energy management strategies. We presented the benefits of strategy use and micro-breaks to the experimental group. Thus, the intervention was provided only to the experimental group. The participants from the control group were contacted twice during the experiment. Therefore, the first contact was two weeks after the first set of questionnaires, when the experimental group had the training. In this first contact, we informed participants in the control group about how many participants we had in the study and thanked them for their participation. The second contact was two weeks before the post-test, in which we reminded them of the next step and thanked them for their contribution. Two weeks after the pre-test (T_1_), the training intervention was delivered online for the entire experimental group by the study’s authors via Microsoft Teams without recording it. At this point, four weeks had passed since the first contact with all the participants in T_1_. The intervention was based on managing physical and mental energy strategies and included a set of steps. The first step was (1) raising awareness of the importance of energy in completing tasks by presenting the definition of PVM and highlighting the benefits of this behavior identified in the literature, such as creativity, job performance, and resilience [29,30].

We presented the context, specifically that work intensity is one of the main demands of the active population in Europe under 25 years and up to 44 years old [31]. Therefore, through energy management, employees could overcome this demand. A second step was (2) presenting the proactive behavior of managing energy as a result of a two-step process: goal setting and goal striving. Steps 1 and 2 represented the introduction. In the next step (3), we presented the definition of energy management strategies; in the fourth step (4), participants identified their energy management strategies by responding to the question: “What strategies do you use to keep you energized while working?”. Participants needed to fill in the behaviors that they considered helpful in managing energy in Mentimeter, a live polling tool for engaging audiences. We aided participants in classifying the suggested behaviors as micro-breaks or work-related strategies. In the next step (5), we presented the energy management strategies identified by previous studies and discussed the extent to which the participants generally used them during working hours. Therefore, steps 3, 4, and 5 shaped the participants’ present state, increasing awareness of the patterns used until the intervention. In the sixth step (6), we presented the importance of the basic rest–activity cycle (90 min of work, 20 min rest) and how they can use it. The basic rest–activity cycle refers to the ultradian rhythms—the biological cycles that repeat throughout a 24 h circadian day. This means that an individual performs best when they work for around 90 min, then take a break for approximately 20 min [32].

Based on the proposed two-step process (i.e., proactive goal generation and proactive goal striving), employees were trained to identify a goal (i.e., task) and then establish the steps to achieve that goal, considering the strategies—this represented step 7. In the last part of the intervention, participants were instructed to identify possible stressors to keep them from taking breaks. Finally, we discussed the most efficient ways to manage these stressors. Steps 6 and 7 aimed to shape the participants’ new approach to managing their energy to function optimally during work. As a final step, we resumed the main ideas from the training. Being the first team to conduct such an intervention, we used a 3-item feedback form to determine the participant’s perception of the activity after the training. The answers were collected right after the training had ended.

**Figure 2:**
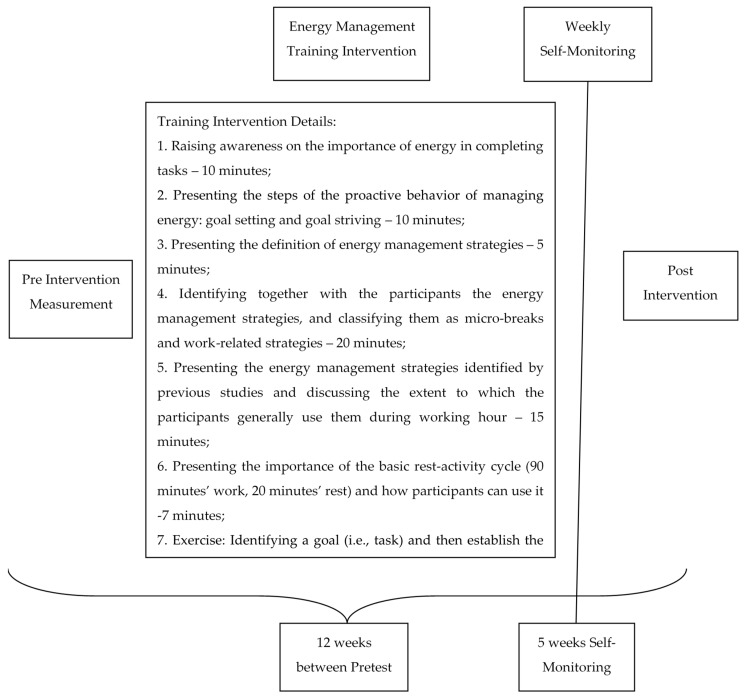
Design of the PVM intervention.

The experimental group started the 5-week self-monitoring phase one week after the training was delivered. Two weeks later, we measured PVM, work-related strategies, and micro-breaks (i.e., mental and physical) in the experimental group at a general level (T2). At the same time, we measured only PVM in the control group. The design of the intervention is illustrated in Figure 2. The entire study took place over 12 weeks during the pandemic. We had a three-month gap between the pre-test and post-test. We expected behavior change to take some time and decided that three months would be relevant [33]. At the same time, we wanted to avoid losing participants due to the length of the study.

### 2.4. Data Analysis

We tested any significant differences in PVM between the intervention and control groups at T_1_ and T_2_. Thus, we conducted a one-way analysis of variance (ANOVA). To test if the training intervention impacted employees’ PVM, we conducted an ANCOVA, including the pre-test as a covariate to control for pre-existing differences in the dependent variable. Furthermore, we tested for differences within the experimental group at T_1_ and T_2_ (pre-test and post-test) using work-related strategies and micro-breaks (i.e., mental and physical) by conducting a paired-sample *t*-test. We used this analysis because we had only two measures, and the *t*-test is more commonly used. To examine whether there were differences in work-related strategies and micro-breaks from the pre-test and the scores from the self-monitoring phase within the experimental group, we conducted a repeated measures ANOVA. All analyses were performed using IBM SPSS Statistics version 23 (SPSS Inc., Chicago, IL, USA). Moreover, following Cohen [34], eta squared in the repeated-measures ANOVA and Cohen’s d as a measure of the effect size for post hoc comparisons were estimated (small effect = 0.2; moderate or medium effect = 0.5; large effect = 0.8). Based on indicators of Skewness and Kurtosis, we considered distributions to be normal, with values ranging from −0.75 to 1.67. Values of Skewness and Kurtosis between −2 and +2 are considered acceptable to prove the presence of a normal univariate distribution [35].

## 3. Results

An analysis of variance was conducted to examine the differences between the experimental and control groups regarding the PVM scores at T_1_ and T_2_. The results showed no differences between the groups at baseline, *F*(1, 84) = 0.38, *p* = 0.53. The results at T_2_ indicated differences between the two groups, *F*(1, 84) = 7.67, *p* = 0.007, with a medium effect size (*d* = 0.59). The results are presented in Table 1.

After the training intervention, an analysis of covariance (ANCOVA) was used to verify differences in PVM between the control and experimental groups. The pre-test value was included as a covariate to control for pre-existing differences in the dependent variable. The results (Table 2) showed that the experimental group scored higher than the control group on PVM, and the difference was significant even when controlling the value of PVM at the pre-test (F = 8.48, *p* < 0.01). Therefore, the results supported Hypothesis 1.

A paired-sample *t*-test was conducted to compare the scores of work-related strategies and micro-breaks (i.e., physical, mental) at T_1_ and T_2_ (or between the pre-test and post-test) within the experimental group. Results are presented in Table 3. There was no significant difference between work-related strategies measured at T_1_ (*M* = 19.07, *SD* = 2.36) and work-related strategies measured after the training intervention at T_2_ (*M* = 17.93, *SD* = 3.84); *t*(41) = 1.66, *p* = 0.10. However, we identified a significant difference in physical micro-breaks from T_1_ to T_2_, *t*(41) = 1.86, *p* = 0.03, with a small effect size (*d* = 0.28). The results regarding mental micro-breaks suggested no significant differences from T_1_ (*M* = 13.95, *SD* = 3.56) to T_2_ (*M* = 13.69, *SD* = 3.46); *t*(41) = 0.35, *p* = 0.36. Thus, the results partially supported Hypothesis 2.

**Figure 3:**
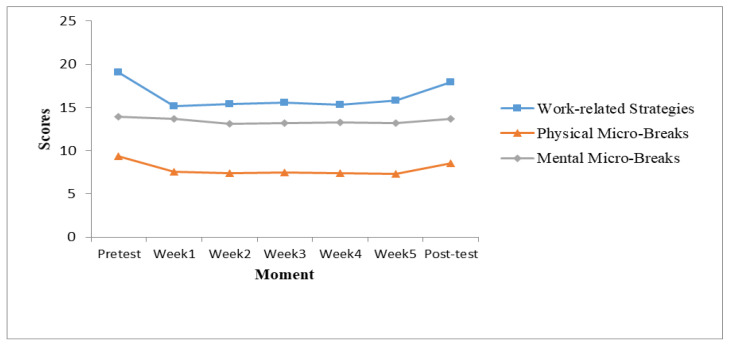
Evolution of the energy management strategies scores during the pre-test, post-test, and the 5-week self-monitoring phase.

Furthermore, a repeated measures ANOVA was conducted to compare the scores of work-related strategies and micro-breaks (i.e., physical, mental) at T_1_ (at pre-test) and the scores from the self-monitoring phase (after weeks 1, 2, 3, 4, and 5). A repeated measures ANOVA (RM ANOVA) with a Greenhouse–Geisser correction indicated that the mean of work-related strategies differed statistically significantly between time points, *F*(3.515, 144.103) = 14.31, *p* < 0.001, *η*^2^ = 0.26). A post hoc analysis with a Bonferroni adjustment revealed that work-related strategies decreased significantly from the pre-test to the monitoring phase, but not from the pre-test to the post-test. Additionally, the RM ANOVA with a Greenhouse–Geisser correction indicated that physical micro-breaks differed significantly between time points, *F*(4.305, 176.525) = 8.35, *p* < 0.001, *η*^2^ = 0.17). The results provided partial support for Hypothesis 2. Effect sizes in the case of work-related strategies were extensive, except for the post-test, ranging from *d* = −0.18 to *d* = −1.18. Effect sizes in the case of physical micro-breaks were medium, with one exception at the post-test, ranging from *d* = −0.28 to *d* = −0.69. The results of RM ANOVA are centralized in Table 4. The post hoc analysis results are centralized in Table 5 and, also, the results are presented in Figure 3.

## 4. Intervention Evaluation

We applied a feedback form to measure the participants’ reactions, i.e., level 1 evaluation; [36]. The results showed high scores on the item reflecting expectations (an average of 4.8 out of 5 points), suggesting that participants’ expectations were met. The same result was obtained for the item that measured the extent to which participants considered the information interesting. An average rating of 4.5 points was recorded on the item, reflecting participants’ opinions on the applicability of the knowledge learned during the intervention. The overall results indicated that participants tended to be very satisfied with the training content.

## 5. Discussion

The central aim of this study was to test whether an intervention using energy management strategies could increase PVM based on the JD-R theory [2]. The results showed that the online training intervention based on these strategies positively impacted PVM scores in the experimental group compared to the control group. Thus, exposing employees to quality information about strategies to manage their energy during work created awareness and changed their energy-management behaviors at work. Additionally, after the initial training, the intervention required a commitment to apply energy management strategies during work for five weeks. In other words, increasing employees’ commitment to the goals assumed at the start of the intervention and receiving quality information about this topic made employees more proactive in managing their energy during work hours [15]. However, looking at the significance of our results regarding the actual use of the proposed energy management strategies, physical micro-breaks are the only ones that showed a slight decrease from the pre-test to the post-test. A similar pattern was identified in reducing work-related strategies in the self-monitoring phase. A possible explanation for these results may be based on individual preferences. Thus, employees may have a better idea of how and when to boost their energy during work. Therefore, employees may use different strategies to manage their physical and mental energy to promote their work than those proposed during the intervention [3].

Moreover, the period between the intervention and the other post-test measurements might have been too short for participants to make substantial changes in their work behavior. This may suggest that an energy management strategy requires sustained exercises to be manifested at work as proactive behavior [37]. Employees who want to manage their cognitive, emotional, and energetic resources must learn and practice different strategies in time.

Nevertheless, we expected a decrease in micro-breaks, as these activities are more related to the recovery process during work [38]. Employees may engage in micro-breaks when their energy level is already low. These actions are contrary to what PVM proposes as an individual strategy. Thus, individuals engage in proactive energy management strategies when their energy is not yet depleted to ensure that it remains at an optimal level. This aligns with the JD-R theory, emphasizing that employees are less likely to use energy management strategies when they are depleted of energy [9]. Additionally, micro-breaks are more likely to be manifested as recovery strategies during work. Moreover, stress may play an essential role in the manifestation of PVM because it depletes the resources needed to initiate these actions. Conversely, we expected an increase in the proposed work-related strategies, but the results only showed small fluctuations during the self-monitoring phase. The main difference between work-related strategies and micro-breaks may be the cognitive effort required. Work-related strategies require more exercise and mental stimulation than micro-breaks that may respond to strain, with strategies oriented toward recovery at work.

An interesting result resides in the change in PVM scores recorded in the control group from pre-test to post-test. The participants in this group seemed to report better energy management at the first measurement compared to the second one. A possible explanation is that, at first, the participants were enthusiastic about the energy management topic. They may have obtained artificially favorable results due to an “enthusiasm effect” [39]. Subsequently, the enthusiasm decreased over time, leading to a lower PVM score. Additionally, participants may have discovered that energy management strategies need mindfulness of one’s energy levels and practice to create a habit. This idea is reflected by the higher scores obtained by the employees exposed to the intervention in the second measurement. The increase is significant, but it also suggests that energy management strategies require practice [9]. Additionally, we observed a general tendency of employees to report high levels of PVM, which indicated that they might overestimate the manifestation of this behavior before knowing more details about it.

A handful of studies have discussed whether employees’ proactive behavior can be facilitated through training [13,15,40]. However, this is the first study to examine the impact of training on PVM. In this regard, we also wanted to measure participants’ reactions to the intervention. Thus, we requested feedback after the training, and the results showed that the participants were delighted with the content and reported applying the learned content. This can be relevant because it offers practitioners an already tested and successful approach.

### 5.1. Theoretical and Practical Implications

Our findings have theoretical implications for the JD-R theory. Specifically, we provide evidence about the role of PVM as an individual strategy for managing energy in the motivational process. From the JD-R perspective, energized employees are more resourceful and protected against burnout, but they should be engaged at work with high vitality. Therefore, through PVM, individuals engage in energy management strategies that promote their health and work-related well-being. We also contribute by differentiating between PVM and other behaviors manifested in response to strain (i.e., recovery behaviors at work). Thus, we broaden the theoretical understanding of these behaviors that help employees to function well at work in different contexts.

A significant practical contribution we make is offering organizations an effective online intervention for developing their employees’ energy management capabilities at work. This is the first intervention designed to enhance PVM. Based on the importance of energy in completing work and previous research, employees’ well-being may be positively impacted by investing in training that targets energy management strategies. In this way, employees can become aware of their health and protect themselves from exhaustion, as they will develop strategies to preserve energy to overcome challenges during work hours. Our intervention also provides an exercise that employees can use to structure their approach in the energy management area. Consequentially, employers’ benefit from business success and engaged employees. Individuals with more personal resources (e.g., energy) and who use self-regulation strategies (e.g., energy management strategies) are more involved in their work because their resources contribute to achieving work goals, which is consistent with the JD-R theory. This is in line with the proposition that personal resources mediate the relationship between job resources and motivational outcomes.

Furthermore, organizations can encourage employees to engage in energy management strategies that best suit their needs. Our results suggest that there are no effective strategies suitable for all individuals. Therefore, organizations should provide the structure and space for taking breaks (e.g., relaxation rooms), and employees will decide how (e.g., helping a co-worker, learning something new, listening to music, navigating the web, making a to-do list) and when is most suitable for them to manage their energy. Moreover, employers should promote a culture where the main focus is on employees’ well-being by positively reinforcing energy management strategies (i.e., work-related, mental, and physical micro-breaks). In the context of the worldwide pandemic, with changes related to work and different plans to return to work (such as hybrid programs and co-working arrangements of the workspace), PVM becomes an excellent individual strategy for employees to increase their energy level during working hours.

### 5.2. Limitations and Future Research

This study has limitations that can raise several ideas for future research. First, our data collection included only self-report measures, which may increase the chances of socially desirable responses. However, this type of measurement is appropriate due to the subjective nature of the measured variables (i.e., PVM, energy management strategies). While self-perceptions are essential in understanding how individuals evaluate their energy management behaviors, the risk of common method bias should not be neglected. Therefore, future research will preferably combine different sources of data for validation (e.g., video data and supervisor feedback on employees’ PVM behavior). By video recording the behavior, more objective data can be obtained. Additionally, another level of objectivity can be achieved by instructing supervisors to record behaviors. Second, energy management behaviors may be manifested unconsciously, and our study did not use measurements to overcome this possibility. For example, employees may engage in a work-related strategy (e.g., making a to-do list) without explicitly aiming to manage their energy. Therefore, it is recommended to use observational methods and measures that are more precise (e.g., record clicking behavior). A third possible limitation is that our study measured PVM and the use of energy management strategies at the pre-test and post-test, complemented by a weekly approach (i.e., every Friday). Therefore, the results may be affected by measuring such a fluctuating resource at wider time intervals without considering hourly changes that could be assessed. Future research should examine PVM and energy management strategies throughout the workday. Fourth, the intervention study occurred during the pandemic, which may have influenced the results. When there is a fine line between working time and personal time, individuals may have overestimated or sometimes underestimated their engagement in PVM and the manifestation of energy management strategies. Fifth, we limited our study to enhancing PVM by only considering energy management strategies. Future research should also use an experimental design to investigate the effect of applied strategies to increase not only PVM but also the outcomes of PVM, such as well-being (e.g., work engagement, mental and physical health, and job satisfaction). The effect of the intervention should be monitored in time, measuring the outcomes also at a one-month follow-up after the intervention. Sixth, our sample was limited to white-collar workers with access to a computer, and we also had a limited number of participants. Future studies require a larger sample than ours to detect significant effects, especially if more variables are included as mediators (e.g., sleep time, physical activity, scholarship level). Therefore, our findings cannot be generalized. Future research should also consider samples that include participants from other occupations.

Choices in using one energy management strategy over another may depend on the task complexity (i.e., high/low) and the timing of the task. Some employees are more active in the morning and may prefer to complete complex tasks at that moment of the day. Additionally, this type of employee may be already energized after an extensive recovery period (i.e., overnight sleep) and is less likely to use energy management strategies. On the other side are employees who are active in the afternoon and who, during the day, try to manage their energy to complete lighter tasks and prepare for the more complex ones. These presumptions are still yet to be investigated and could be a good idea for new research. Finally, our study can be considered a pilot study as it is the first to investigate the increase in PVM based on specific online intervention. Future research can use the present results as a starting point and develop more complex designs to examine other variables by interacting with PVM.

## 6. Conclusions

Our study focuses on the energy of individuals and whether it can be better managed during work. The results showed that an online training intervention based on energy management techniques positively impacted employees’ PVM. Energy management strategies in the workplace are one of the handiest individual strategies organizations can develop among their employees through training. Furthermore, PVM, as a self-regulation strategy, is a proactive behavior that is the responsibility of each employee because it is related to individual needs, and the constantly changing context requires more and more proactive employees.

## Figures and Tables

**Table 1:** ANOVA Results for PVM at Time 1 and Time 2.

		Control Group (N = 44)		Experimental Group (N = 42)					
Variable	Time	M	SD	M	SD	F	df	*p*	d
PVM	1	46.02	4.47	45.31	6.06	0.38	1	0.53	-
	2	41.59	9.85	46.36	5.33	7.67 *	1	0.007	0.59

* Note: PVM = Proactive vitality management.

**Table 2:** ANCOVA results for post-PVM differences.

Scale	Group						df	F	*p*	d
	Control (N = 44)			Experimental (N = 42)						
	EM	M	SD	EM	M	SD				
PVM	41.49	41.59	9.85	46.45	46.36	5.33	1	8.48 *	0.005	0.59
Pre-test							1	2.96	0.089	-

* Notes: PVM = Proactive vitality management, EM = Estimated mean.

**Table 3:** Paired *t*-test results for the comparison of work-related strategies and micro-breaks (i.e., physical, mental) at the pre-test and post-test within the experimental group.

Variable	Pre-Test		Post-Test		df	*t*-Test	*p*	d
	M	SD	M	SD				
Work-related strategies	19.07	2.36	17.93	3.84	41	1.66	0.104	-
Physical micro-breaks	9.33	2.76	8.55	2.53	41	1.86	0.034	0.28
Mental micro-breaks	13.95	3.56	13.69	3.46	41	0.35	0.363	-

**Table 4:** Repeated measures ANOVA for the comparison of work-related strategies and micro-breaks (i.e., physical, mental) at the pre-test versus self-monitoring moments and post-test within the experimental group.

Variable	F	df	*p*	η^2^
Work-related strategies	14.31	3.5, 144.1	<0.001	0.26
Physical micro-breaks	8.35	4.31, 176.52	<0.001	0.17
Mental micro-breaks	0.72	3.34, 136.72	0.556	0.02

**Table 5:** Post hoc comparison between measurement moments of work-related strategies and micro-breaks (i.e., physical, mental) at the pre-test versus self-monitoring moments and post-test within the experimental group.

Variables	Time	M	SD	MD	*p*	d
Work-related strategies	Pre-test	19.07	2.36			
	Week 1	15.19	2.99	3.88	<0.001	−0.97 [−1.44, 0.50]
	Week 2	15.40	2.95	3.66	<0.001	−1.18 [−1.66, −0.70]
	Week 3	15.57	3.54	3.50	<0.001	−0.98 [−1.45, −0.50]
	Week 4	15.31	3.22	3.76	<0.001	−0.81 [−1.28, −0.35]
	Week 5	15.81	3.84	3.26	<0.001	−0.89 [−1.36, −0.42]
	Post-test	17.93	3.84	1.14	1.00	−0.18 [−0.61, 0.25]
Physical micro-breaks	Pre-test	9.33	2.76			
	Week 1	7.57	2.38	1.76	0.005	−0.57 [−1.00, −0.13]
	Week 2	7.36	2.12	1.97	0.001	−0.63 [−1.07, −0.20]
	Week 3	7.45	2.32	1.88	0.001	−0.69 [−1.12, −0.25]
	Week 4	7.40	2.16	1.93	0.001	−0.64 [−1.08, −0.20]
	Week 5	7.31	2.01	2.20	<0.001	−0.67 [−1.12, −0.23]
	Post-test	8.55	2.53	.78	1.00	−0.28 [−0.71, 0.15]
Mental micro-breaks	Pre-test	13.95	3.56			
	Week 1	13.67	3.58	.28	1.00	−0.10 [−0.53, 0.33]
	Week 2	13.14	3.46	.81	1.00	−0.29 [−0.72, 0.14]
	Week 3	13.19	3.89	.76	1.00	−0.26 [−0.69, 0.17]
	Week 4	13.29	3.18	.66	1.00	−0.20 [−0.62, 0.23]
	Week 5	13.21	3.41	.73	1.00	−0.22 [−0.65, 0.21]
	Post-test	13.69	3.46	.26	1.00	−0.04 [−0.47, 0.39]

Notes: MD = mean difference; mean difference is between the pre-test and the other moments (week 1, 2, 3, 4, and post-test).
